# Combining Bioinformatics and Experiments to Identify and Verify Key Genes with Prognostic Values in Endometrial Carcinoma

**DOI:** 10.7150/jca.35854

**Published:** 2020-01-01

**Authors:** Wenchao Zhang, Lingling Gao, Caixia Wang, Shuang Wang, Di Sun, Xiao Li, Miao Liu, Yue Qi, Juanjuan Liu, Bei Lin

**Affiliations:** 1 Department of Obstetrics and Gynaecology, Shengjing Hospital Affiliated to China Medical University, Liaoning, China,.; 2 Key Laboratory of Maternal-Fetal Medicine of Liaoning Province, Key Laboratory of Obstetrics and Gynecology of Higher Education of Liaoning Province, Liaoning, China.

**Keywords:** GEO, TCGA, UBE2C, endometrial carcinoma, prognosis

## Abstract

Endometrial carcinoma(EC) is the most common cancer of female reproductive system, thus requiring for new effective biomarkers which could predict the onset of EC and poor prognosis. Our study integrated two GEO datasets(i.e.GSE63678, GSE17025) and TCGA(The Cancer Genome Atlas ) UCEC data to screen out 344 common differentially expressed genes(DEGs), which were further analyzed by GO(gene ontology) functions and KEGG(Kyoto Encyclopedia of Gene and Genome) pathways. KEGG analysis results showed these DEGs were mainly enriched in cell cycle, oocyte meiosis, cellular senescence, carbon metabolism and p53 signaling pathway. Top 20 hub genes with higher degree were selected from PPI(protein-protein interaction) network and 15 of them were associated with the prognosis of EC, that is, CCNB2, CDC20, BUB1B, UBE2C, AURKB, FOXM1, NCAPG, RRM2, TPX2, DLGAP5, CDCA8, CDC45, MKI67, BUB1, KIF2C. UBE2C(Ubiquitin Conjugating Enzyme E2 C) was chosen for further validation in TCGA cohort on mRNA level and in our patient samples on protein level by immunohistochemistry. UBE2C was significantly highly expressed in endometrial carcinoma, and its expression level was associated with advanced FIGO staging and poor prognosis. Cox risk model demonstrated high UBE2C expression was an independent risk factor. Somatic mutations, elevated copy number, DNA hypomethylation all contributed to its overexpression. Therefore, by combination of bioinformatics and experiment, our study provided a unique insight into the pathogenesis and molecular mechanisms underlying EC and discovered new biomarkers for early diagnosis and prognostic prediction. UBE2C could serve as a potential marker to predict poor prognosis and as a therapeutic target.

## Introduction

Endometrial carcinoma(EC) is the most common cancer in female reproductive system in the United States^1^ and the second most common cancer worldwide, only after cervical cancer^2^. In some socioeconomic transitioning countries, the incidence rate is still on the rise^3^. Postmenopausal abnormal vaginal bleeding can be early diagnostic sign in EC patients, but most women with the sign will not be diagnosed with endometrial carcinoma^4^.

EC is divided into two subtypes, estrogen dependent subtype (type I) and gene mutation-related subtype (type II). Type I EC is classified as endometrioid adenocarcinoma, the most frequently occurring histological subtype, which usually has a better prognosis. Type II endometrial cancer is described as non-endometrioid, mutant gene (P53,P16, etc.)harbored, which is associated with a higher risk of metastasis and a poor prognosis^5^.

In most cases, patients are diagnosed as stage I~II(International Federation of Gynecology and Obstetrics [FIGO] stages) carcinomas, who will have a better prognosis with 5-year overall survival rate ranging from 74%~91%. However, some endometrial cancers may reach an advanced stage before signs and symptoms can be noticed, as is seen that stage III~IV patients have a worse prognosis and their 5- year overall survival rates stand roughly at 57~66% and 20~26%, respectively^6^. The lack of early diagnostic and therapeutic biomarkers is held responsible for most deaths caused by EC.

Nowadays, the exceptional heterogeneity of cancer and individual differences pose extra difficulty to its diagnosis and precision treatment. As the application of high throughout sequencing infiltrates into clinical studies, large volumes of patient information are available online. Till now, no research has focused on screening for new prognostic biomarkers in endometrial carcinoma. Therefore, our study aimed to identify potential key genes with prognostic significance by bioinformatic method and advance our understanding of the tumorigenesis and progression of EC. We then integrated two GEO endometrial carcinoma datasets and TCGA UCEC(uterine corpus endometrial carcinoma) cohort to detect common differentially expressed genes(DEGs) , which were further analyzed by gene ontology(GO) functions and Kyoto Encyclopedia of Gene and Genome (KEGG) pathways.

Then, PPI (protein-protein interaction) network was built using the Search Tool for the Retrieval of Interacting Genes (STRING) database to select hub genes with higher degree. 15 hub genes with prognostic predictive potential out of top 20 were verified in TCGA cohort on mRNA level, one of which was UBE2C. The ubiquitin-dependent proteolysis is an important cellular mechanism for targeting abnormal or short-lived proteins for degradation. Human UBE2C(Ubiquitin Conjugating Enzyme E2 C) gene is mapped to chromosome 20q13.12 and encodes a member of the E2 ubiquitin-conjugating enzyme family, which is required for the destruction of mitotic cyclins and for cell cycle progression^7^. Recent literature has reported that UBE2C plays critical role in the progression of non-small cell lung cancer 8, gastric cancer 9 and breast cancer^10^. Only one article reported that UBE2C expression in endometrial carcinoma group was significantly higher than that in benign and hyperplastic tissues 11. However, the association of its expression level and prognosis has not been investigated. We then carried out immunohistochemistry in more than 100 patient samples to validate UBE2C expression on protein level. The results demonstrated that UBE2C expression was significantly correlated with FIGO staging and lymphatic metastasis. UBE2C could act as an independent predictor for the prognosis of endometrial carcinoma.

## Materials and methods

### Microarray data

Two gene expression profile matrix files, namely, GSE63678 12, GSE17025 13 were downloaded from the GEO database. GSE63678 included 7 endometrial cancer tissue/cell and 5 normal endometrial tissue/cells. GSE17025 included 91 endometrial carcinoma tissues and 12 non-cancerous samples. The platforms of these two datasets were GPL571([HG-U133A_2] Affymetrix Human Genome U133A 2.0 Array), GPL570([HG-U133_Plus_2] Affymetrix Human Genome U133 Plus 2.0 Array), respectively. The probes were converted into the corresponding gene symbol according to the annotation information on the platform. All gene expression data were subjected to log2 transformation.

### TCGA RNA-sequencing patient data

The gene expression data (575 cases, Workflow Type: HTSeqCounts) were downloaded from The Cancer Genome Atlas(TCGA) official website for the Uterine Corpus Endometrial Carcinoma projects (UCEC). It included 23 normal endometrial specimens, 543 endometrial carcinoma specimens, with 9 repeated cancerous ones excluded.

Clinical information of the patients, gene-level copy number variation (CNV) profile and gistic2 thresholded analyzed by the GISTIC2.0^14^ method and somatic non-silent mutation (gene-level) were acquired from the website of UCSC Xena.

Details of the aforementioned 3 datasets were shown in Table 1.

### Data processing and screening for DEGs

The limma 15 R package was used to screen for DEGs in GSE63678, GSE17025 datasets, in which genes with P-value < 0.05 and |log fold change (FC)| > 1 were considered DEGs. The DESeq2 R package was used to discover DEGs in TCGA UCEC RNA-sequencing data, in which genes with P-value < 0.05 and |log fold change (FC)| > 1 were considered DEGs. Venn diagram was used to obtain DEGs among those 3 datasets.

### GO and KEGG enrichment analyses of DEGs

The Database for Annotation, Visualization and Integrated Discovery (DAVID) 16 (version 6.8) provides a comprehensive set of functional annotation tools for investigators to understand biological meaning behind large list of genes. GO functional annotation and KEGG of DEGs was analyzed and visualized by clusterProfiler.R package 17.

### PPI network construction and key module identification

The PPI network was built using Search Tool for the Retrieval of Interacting Genes (STRING) (version 10.0)^18^ online database. Cytoscape (version 3.6.1) is a bioinformatic software for visualizing molecular interaction networks. The plug-in Molecular Complex Detection (MCODE) of Cytoscape is an APP to find closely connected regions in a network 19. The PPI network was visualized using Cytoscape and the most significant module was identified using MCODE. The selection criteria were: degree cut-off=2, node score cut-off=0.2, Max depth=100 and k-score=2.

### Prediction of prognostic significance

TCGA RNA-sequencing patient data was used to screen for genes with prognostic predictive potential out of the top 20 hub genes with higher degree(the number of direct connections that a node has with other nodes). Survminer R package was used to detect a value of a cutpoint that corresponds to the most significant relation with outcome (here, survival probability).

### cBioportal data extraction

cBioportal (TCGA Uterine Corpus Endometrial Carcinoma, n = 548) 20 was utilized to extract the data of DNA methylation of UBE2C and its mRNA expression. Co-expressed genes with UBE2C were also downloaded from cBioportal.

### Gene set enrichment analysis (GSEA)

TCGA patients were divided into high- and low-UBE2C phenotypes. The cutpoint was 11.49, which was the same as the one of survival probability curve. GSEA 3.0 software 21 was used to analyze the data. h.all.v6.2.symbols.gmt[Hallmarks] was set as the gene set database, gene set permutations as 1,000 times. Enrichment analysis was performed between the two groups using default weighted enrichment statistics.

### Participants and specimens

Endometrial carcinoma, atypical hyperplasia endometrium and normal endometrium samples were collected from 129 surgical patients at the Department of Gynecology from Shengjing hospital from 2007 to 2013. All the patients were informed of the experiments and signed informed consent was obtained. The tissue-associated experiments were approved by the Clinical Research Ethics Committee of Shengjing Hospital affiliated to China Medical University. Samples were embedded in paraffin and all the diagnoses of the pathological sections were made by experienced pathologists. The median ages of the patients at diagnosis who offered samples of proliferative phase of normal endometrium, secretory phase of normal endometrium, mild atypical hyperplasia, moderate atypical hyperplasia, severe atypical hyperplasia, and endometrial carcinoma were 43 (38-53), 44 (23-58), 43.5(36-49), 41 (30-66),51(38-55) and 58(36-79) years old, respectively. No significant difference(p>0.05) was noted among those groups.

The 129 specimens included 34 cases of normal endometrium (14 proliferative,20 secretory), 23 cases of atypical hyperplasia (8 mild atypical hyperplasia,9 moderate atypical hyperplasia, 6 severe atypical hyperplasia) and 72 cases of endometrial carcinoma.

Patient characteristics of our samples were listed in Table 2.

All tumors originated from the primary site, with no radiotherapy, chemotherapy, or hormone therapy treated before surgery.

All procedures performed in the studies involving human participants were in line with the ethical standards of the institutional and/or national research committee and with the 1964 Helsinki Declaration and its later amendments or comparable ethical standards.

### Immunohistochemistry

Each specimen was fixed in 10% formalin, embedded in paraffin blocks, and processed as continuous sections (5 µm thick). Specimens were dewaxed by discontinuous concentrations of ethanol and blocked to deactivate endogenous peroxidase for 25min. They were then heated in a microwave in citrate buffer solution to retrieve antigens for 18 mins, cooled to room temperature, and blocked by incubation in goat serum for 25 minutes at 37°C. Sections were incubated in rabbit anti-UBE2C antibody(Abcam, Cambridge, UK; 1:100) overnight at 4°C, followed by incubation with horseradish peroxidase-coupled goat anti-rabbit secondary antibody at 37°C for 35 minutes, and stained by 3,3′-diaminobenzidine. The nucleus was counterstained blue by hematoxylin. Sections were then dehydrated, cleared by xylene, and mounted. UBE2C expression was detected by streptavidin peroxidase method. For each batch, breast cancer sample was used as positive control. The samples incubated with PBS instead of UBE2C primary antibody were used as negative control. The experimental procedure was performed strictly following the manufacturer's instructions.

### Assessment of immunohistochemical staining

Five fields under the microscope at 400× magnification were randomly selected and scored. The result was considered positive if yellow staining was found in the cytosol and membrane. Staining was classified as negative, light yellow, brownish yellow, and dark brown, which were scored 0, 1, 2, or 3, respectively. The percentage of positive cells in a field of view under the microscope was assessed and scored 0, 1, 2, 3, or 4 if the percentage was <5%,5% ~25%, 26% ~50%, 51% ~75%, and >75%, respectively. The final result of each specimen was calculated by the product of those two scores. The staining was considered negative (-), weak positive (+), positive (++), and strong positive (+++) if the product fell between 0 and 2, 3 and 4, 5 and 8, and 9 and 12, respectively. In order to decrease errors, every field was read by two observers independently and would be judged by another person when the results were inconsistent.

### Statistical analysis

The analysis of GEO and TCGA databases was conducted using R (v.3.5.1) language. The relationship between clinical pathologic features and UBE2C were analyzed by Students' t test and logistic regression. The influence of its expression on the UCEC overall survival was analyzed by Kaplan-Meier method. In our patient samples, χ2 test was adopted to analyze the relationship of UBE2C expression and clinicopathologic characteristics using SPSS software (version 22). Univariate cox regression and the Kaplan-Meier method were used to analyze the impact of UBE2C expression and clinicopathologic parameters on the overall survival of the patients. Multivariate Cox analysis was employed to compare the impact of UBE2C expression on survival along with other clinical characteristics (stage, grade, myometrial invasion, lymphatic metastasis and age).

## Results

### Identification of DEGs

GSE63678 dataset contained 1011 deferentially expressed genes, of which 536 were up-regulated ,475 down-regulated (Table S1). GSE17025 dataset contained 2715 deferentially expressed genes, of which 1023 were up-regulated, 1692 down-regulated (Table S2). In TCGA UCEC dataset, 3777 up-regulated genes and 2556 down-regulated genes were detected (Table S3).

**Figure 1:**
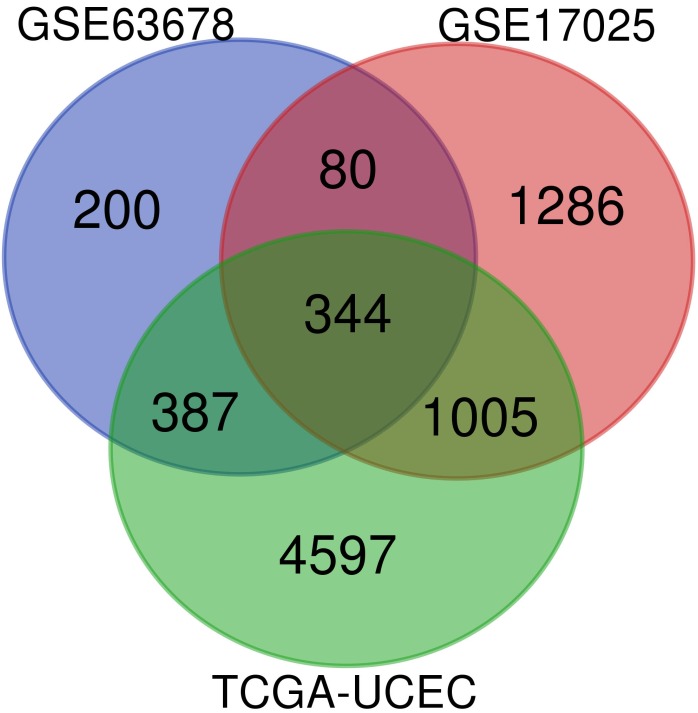
Identification of DEGs: venn diagram of GSE63678, GSE17025 and TCGA-UCEC

Venn diagram was used to screen for common DEGs among those 3 aforementioned datasets, as shown in Figure 1. As a result, 344 DEGs were discovered, among which 170 genes were up-regulated, 174 genes were down-regulated.

**Figure 2:**
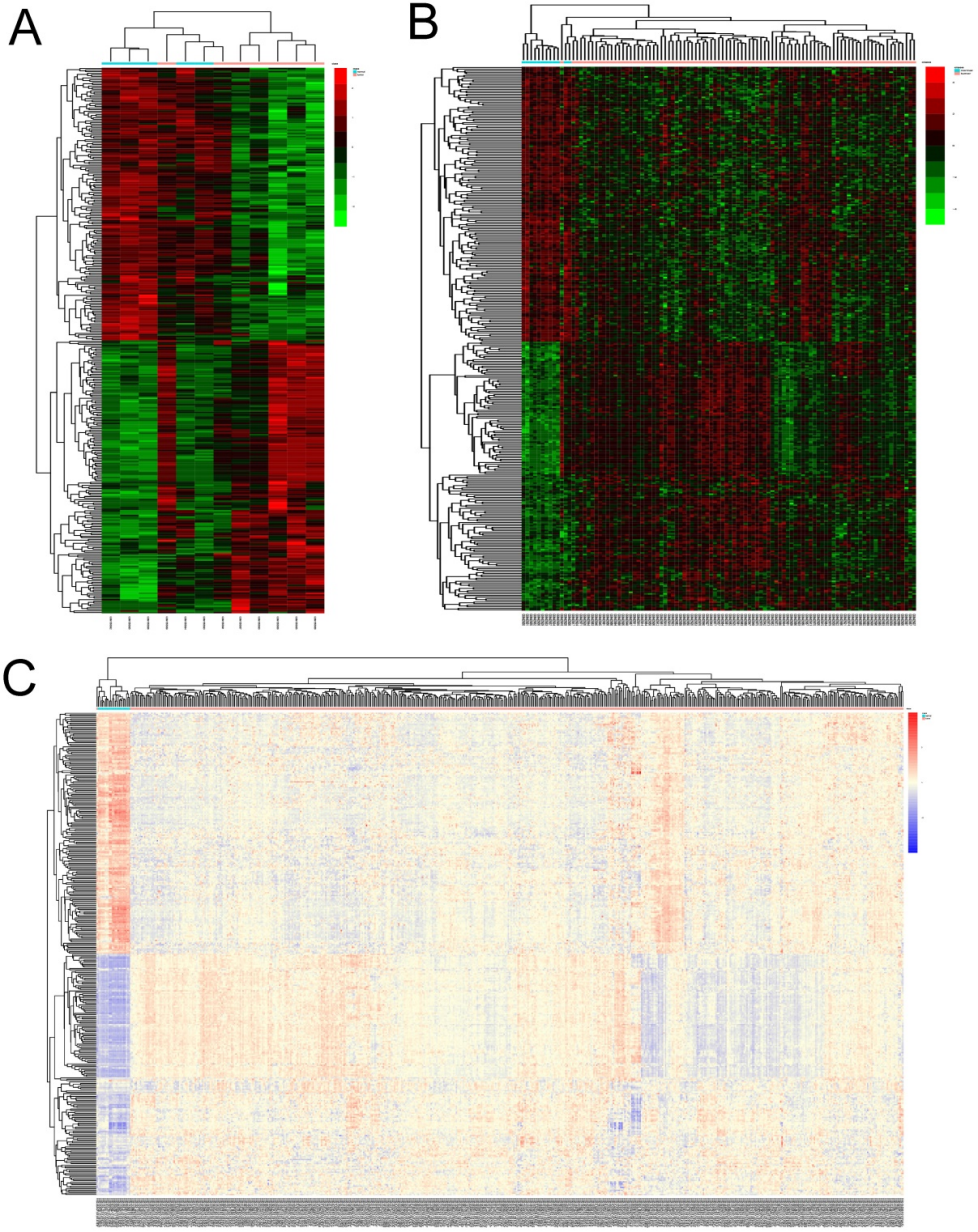
** Heatmaps of DEGs in different datasets. (A)** heatmap of differentially expressed genes(DEGs) in GSE63678. **(B)**heatmap of DEGs in GSE17025. Red represents relative upregulation of gene expression; green represents the relative downregulation of gene expression; black represents no significant change in gene expression. **(C)**heatmap of DEGs in TCGA-UCEC. Red represents relative upregulation of gene expression; blue represents the relative downregulation of gene expression; yellow represents no significant change in gene expression.

The expression profiles of the 344 DEGs were extracted from the 3 datasets and were displayed by cluster heatmaps, as is shown in Figure 2. Sample clustering was completed, with blue representing normal samples and red tumor samples annotated at the top of each plot.

### GO functional enrichment analysis of DEGs

The GO functional enrichment analysis of DEGs was divided into three parts: biological process (BP), molecular function (MF) and cellular component (CC). The results were considered statistically significant if P<0.05.

As is demonstrated in Table 3, the upregulated genes were mainly enriched in mitotic nuclear division (ontology: BP), the spindle (ontology: CC), and microtubule binding(ontology:MF) and the downregulated genes were mainly enriched in positive regulation of developmental growth (ontology: BP), extracellular matrix (ontology: CC) and calcium channel activity (ontology: MF).

### KEGG pathway analysis of DEGs

As is shown in Table 4, the upregulated DEGs were mainly enriched in five pathways, that is, cell cycle (hsa04110), oocyte meiosis (hsa04114), cellular senescence (hsa04218), carbon metabolism (hsa01200), p53 signaling pathway (hsa04115).The downregulated DEGs were mainly enriched in five pathways, namely, EGFR tyrosine kinase inhibitor resistance (hsa01521), JAK-STAT signaling pathway (hsa04630), Calcium signaling pathway (hsa04020), Melanoma (hsa05218), MicroRNAs in cancer (hsa05206).

### PPI network construction and identification of hub genes

**Figure 3:**
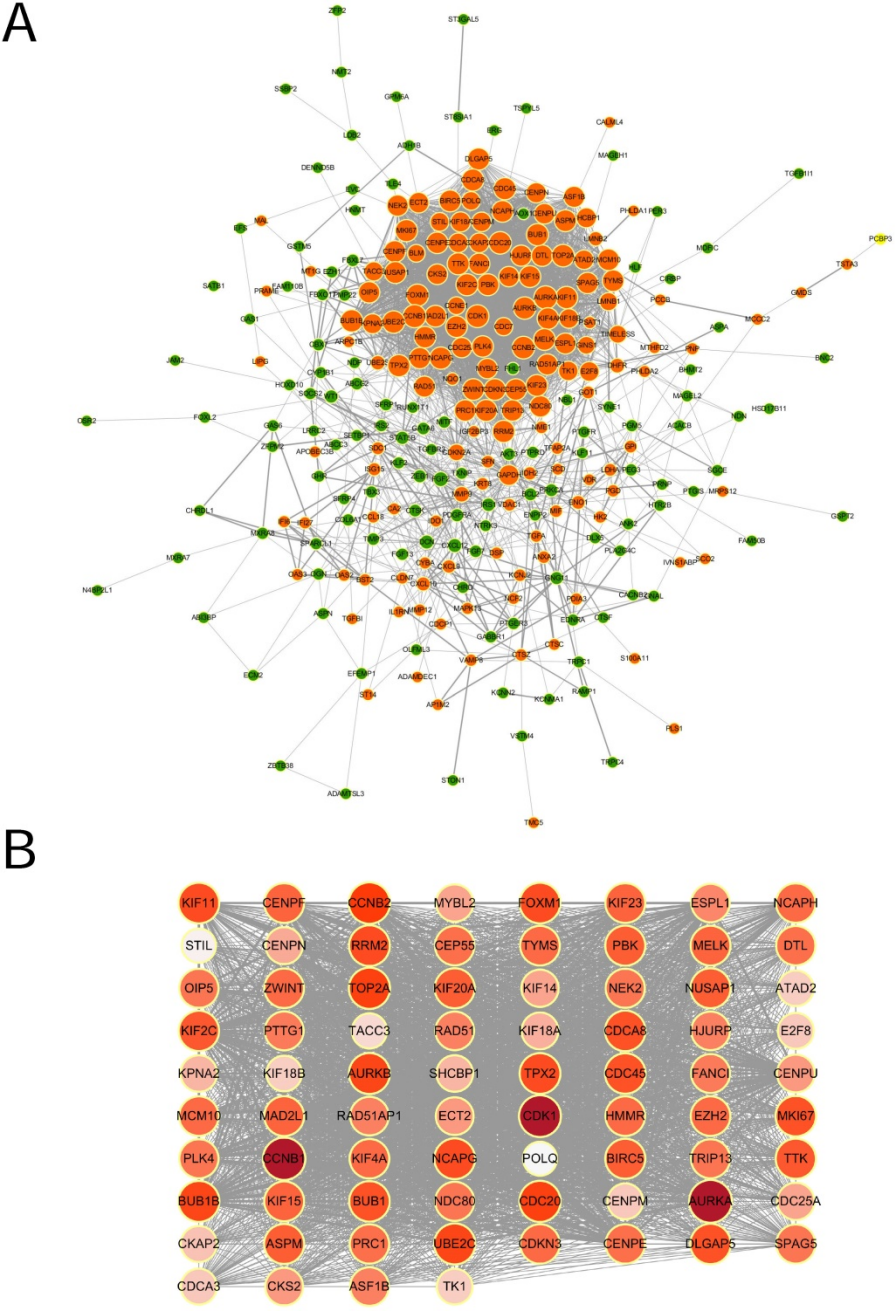
** PPI network of DEGs and the module identified by MCODE. (A)** Protein-protein interaction network of DEGs. Each node represented a protein. Orange nodes were upregulated ones, green downregulated ones. The size of the nodes was proportional to the degree of the nodes. The width of the edges was proportional to the score of protein-protein interaction. **(B)** The significant densely-connected module identified by MCODE plug-in. The color shade of the nodes was proportional to the degree of the nodes.

The STRING online database was used to analyze the interactions among the DEGs. The results were extracted and visualized using Cytoscape software. After excluding the isolated nodes, the final PPI network, as is shown in Figure 3A, was composed of 284 nodes and 3697 edges.

A significant densely-connected module was identified by MCODE plug-in, which had 76 nodes and 2677 edges (Figure 3B). The clustering coefficient was 0.952. We selected top 20 hub genes with higher degree(the number of direct connections that a node has with other nodes) to investigate their effect on the prognosis, which were AURKA (Aurora Kinase A), CCNB1 (Cyclin B1), CDK1 (Cyclin Dependent Kinase 1), CCNB2(Cyclin B2), CDC20(Cell Division Cycle 20), TOP2A(DNA Topoisomerase II Alpha), BUB1B (BUB1 Mitotic Checkpoint Serine/Threonine Kinase B), UBE2C (Ubiquitin Conjugating Enzyme E2 C), AURKB (Aurora Kinase B), FOXM1 (Forkhead Box M1), NCAPG (Non-SMC Condensin I Complex Subunit G), KIF11 (Kinesin Family Member 11), RRM2 (Ribonucleotide Reductase Regulatory Subunit M2), TPX2(Microtubule Nucleation Factor), DLGAP5 (DLG Associated Protein 5), CDCA8 (Cell Division Cycle Associated 8), CDC45 (Cell Division Cycle 45), MKI67 (Marker Of Proliferation Ki-67), BUB1 (BUB1 Mitotic Checkpoint Serine/Threonine Kinase) and KIF2C (Kinesin Family Member 2C).

### Survival analysis of hub genes in TCGA

**Figure 4:**
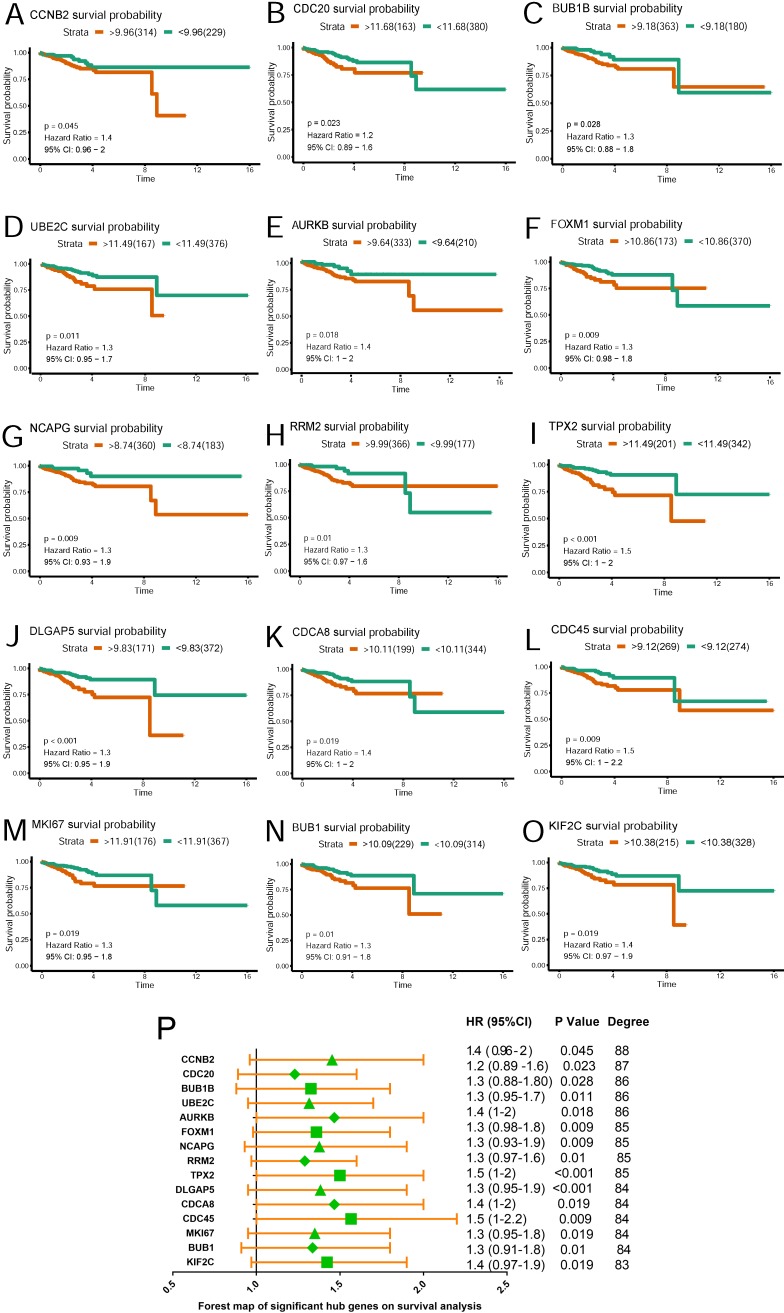
** Survival plot of 15 hub genes and forest map of significant hub genes on survival analysis**. **(A~O)** Survival K-M plotter of significant hub genes with higher degree values. Red lines represented higher expression of the gene, green ones lower expression. X axis means survival time(year). Y axis means survival probability. A,CCNB2. B,CDC20. C,BUB1B. D,UBE2C. E,AURKB. F,FOXM1. G,NCAPG. H,RRM2. I,TPX2. J,DLGAP5. K,CDCA8. L,CDC45. M,MKI67. N,BUB1. O,KIF2C. **P.** Forest map of significant genes on survival analysis.

Based on TCGA data, Kaplan-Meier plots were drawn to reveal the effect of the top 20 hub genes on survival probability. As our results showed in Figure 4 (A~O), 15 genes out of 20 were found to be significantly associated with prognosis in EC patients(p<0.05). EC patients with lower expression of the 15 genes had a better prognosis than those with higher expression. Hazard ratio and confidence interval were also calculated. Forest map(Figure 4P) was produced to compare the differences of those hub genes.

A hub gene UBE2C was selected for further validation for its higher degree value(Degree=86) and lower p value(p=0.011), which we believe played critical role in the PPI network.

### Validation of UBE2C in TCGA

**Figure 5:**
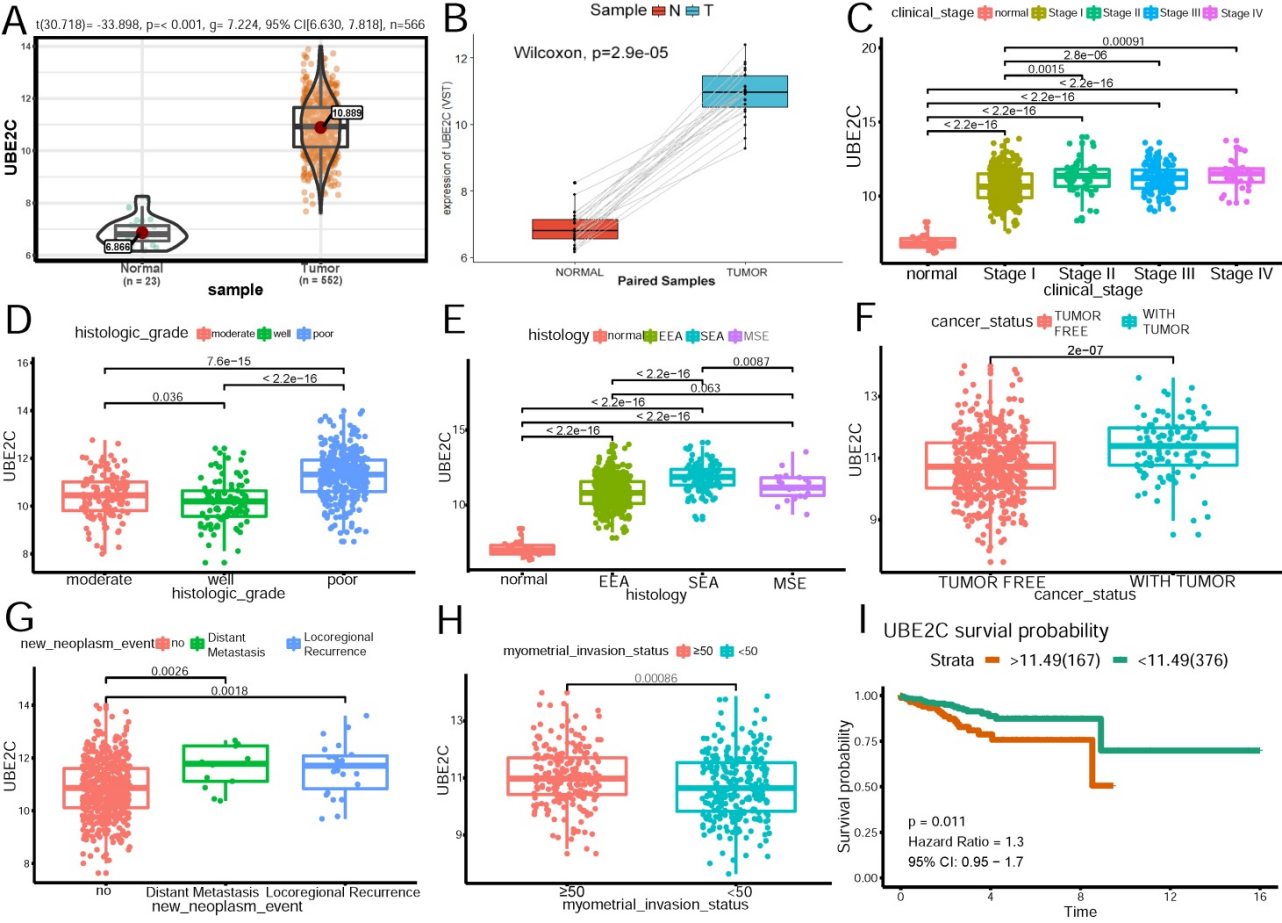
** The relationship of UBE2C expression and clinicopathologic parameters. (A)**Expression level of UBE2C in normal and tumor samples. **(B)** Expression level of UBE2C in paired samples. **(C)**Association of UBE2C expression with clinical stage. **(D)**Association of UBE2C expression with histologic grade. **(E)**Association of UBE2C expression with histologic type. EEA: endometrioid endometrial adenocarcinoma. SEA: serous endometrial adenocarcinoma. MSE: mixed serous and endometrioid adenocarcinoma. **(F)**Association of UBE2C expression with cancer status. **(G)**Association of UBE2C expression with distant metastasis and locoregional recurrence. **(H)** Association of UBE2C expression with myometrial invasion status. **(I)** The effect of UBE2C expression on the overall survival in endometrial patients. Time was calculated by year.

UBE2C was chosen for further analysis on the relationship between its expression and clinicpathological parameters in TCGA cohort. Survival curve(Figure 5I) of the gene was also generated using TCGA data. The best cutpoint of the UBE2C expression was 11.49.

As Figure 5A shows, UBE2C expression was significantly higher in tumor patients than that in normal tissues(p<0.001). To shore up the evidence, cancerous samples were matched with their normal ones(Figure 5B), which was consistent with the former conclusion. As is demonstrated in Figure 5 C~H, increased expression of UBE2C was significantly associated with the advanced FIGO stage(stage I vs stage II~IV) (p=0.0015, p=2.8e-06, p=0.00091, respectively), poor differentiation (p<0.05), serous endometroid carcinoma (p <0.001), with-tumor status (p<0.001), ≥50% myometrial invasion (p=0.00086) and distant metastasis as well as locoregional recurrence(p = 0.0018, p=0.0026, respectively).

Univariate analysis using logistic regression revealed that high UBE2C expression as a categorical independent variable (based on the optimal cutpoint 11.49) was associated with poor prognostic clinicpathologic characteristics (Table 5). Overexpressed UBE2C in EC was significantly correlated with advanced FIGO stage (OR = 3.152 for stage IV vs. stage I), high grade(OR = 7.578 for poor vs. well), histology (OR = 4.813 for serous vs. endometrioid), distant metastasis (OR = 4.043 for positive vs. negative), with-tumor status (OR=2.548 for with-tumor status vs tumor-free status) (all p-values < 0.05), except deeper myometrial invasion(p>0.05).

These results indicated that patients with high UBE2C expression tended towards a more advanced stage and poor prognosis than those with low UBE2C expression.

### GSEA identified UBE2C-related signaling pathways

To explore and identify the potential function of UBE2C in EC, GSEA was conducted between the high- and low-UBE2C expression groups.

**Figure 6:**
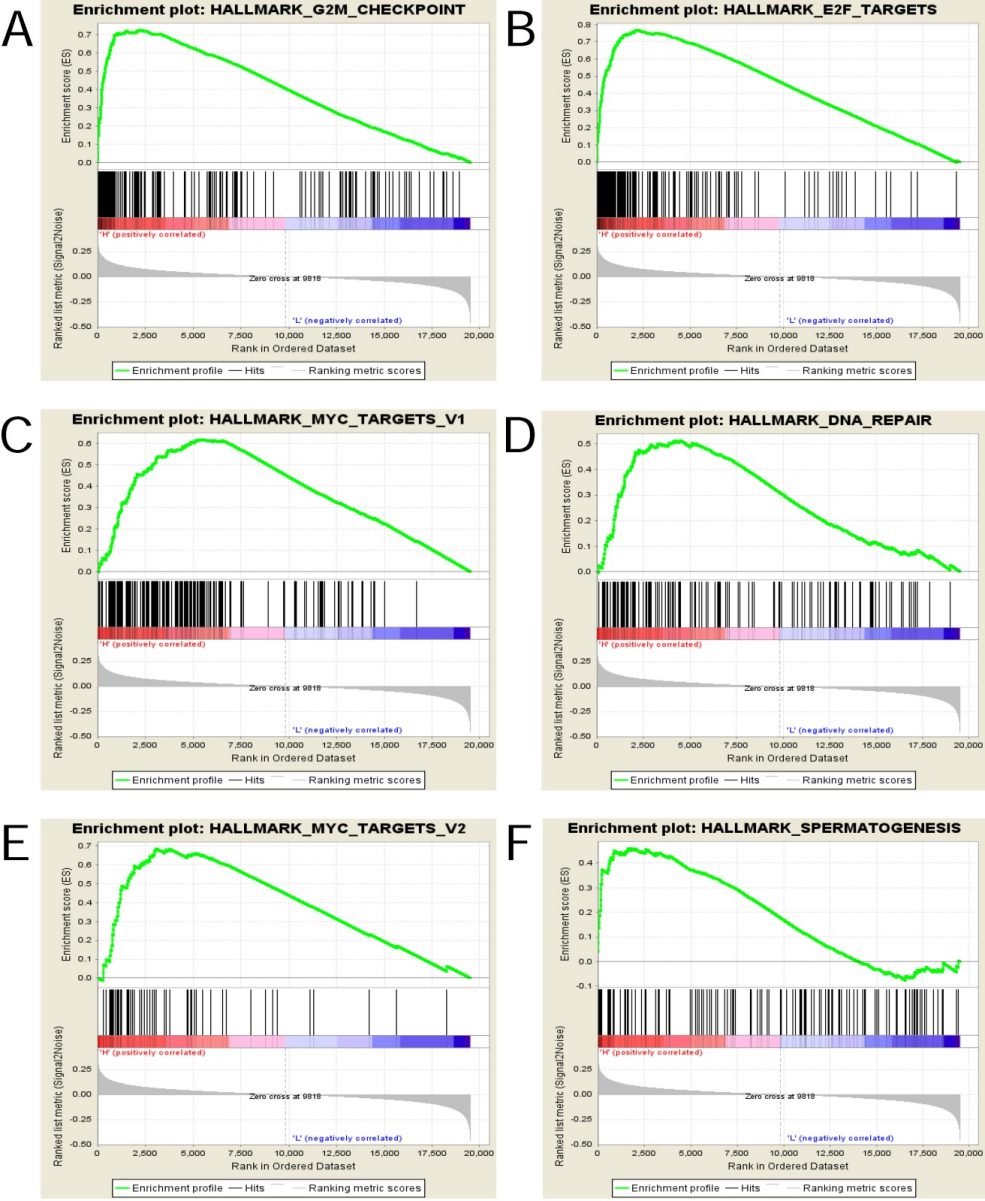
** Enrichment plots from gene set enrichment analysis (GSEA). (A)**G2M checkpoint **(B)**E2F targets **(C)**MYC targets v1 **(D)** DNA repair **(E)** MYC targets v2 and **(F)**Spermatogenesis were differentially enriched in highly expressed group of UBE2C.

The gene sets with the nominal pvalue<0.05 and FDR<0.25 were considered significantly enriched and the top 6 in phenotype high(n = 167) were displayed in Figure 6 and Table 6, that is , “HALLMARK_G2M_CHECKPOINT”, “HALLMARK_E2F_TARGETS”, “HALLMARK_MYC_TARGETS_V1”, “HALLMARK_DNA_REPAIR”, “HALLMARK_MYC_TARGETS_V2” and “HALLMARK_SPERMATOGENESIS” .

### Validation of UBE2C expression in our patient samples

#### UBE2C expression in different groups of our samples

**Figure 7:**
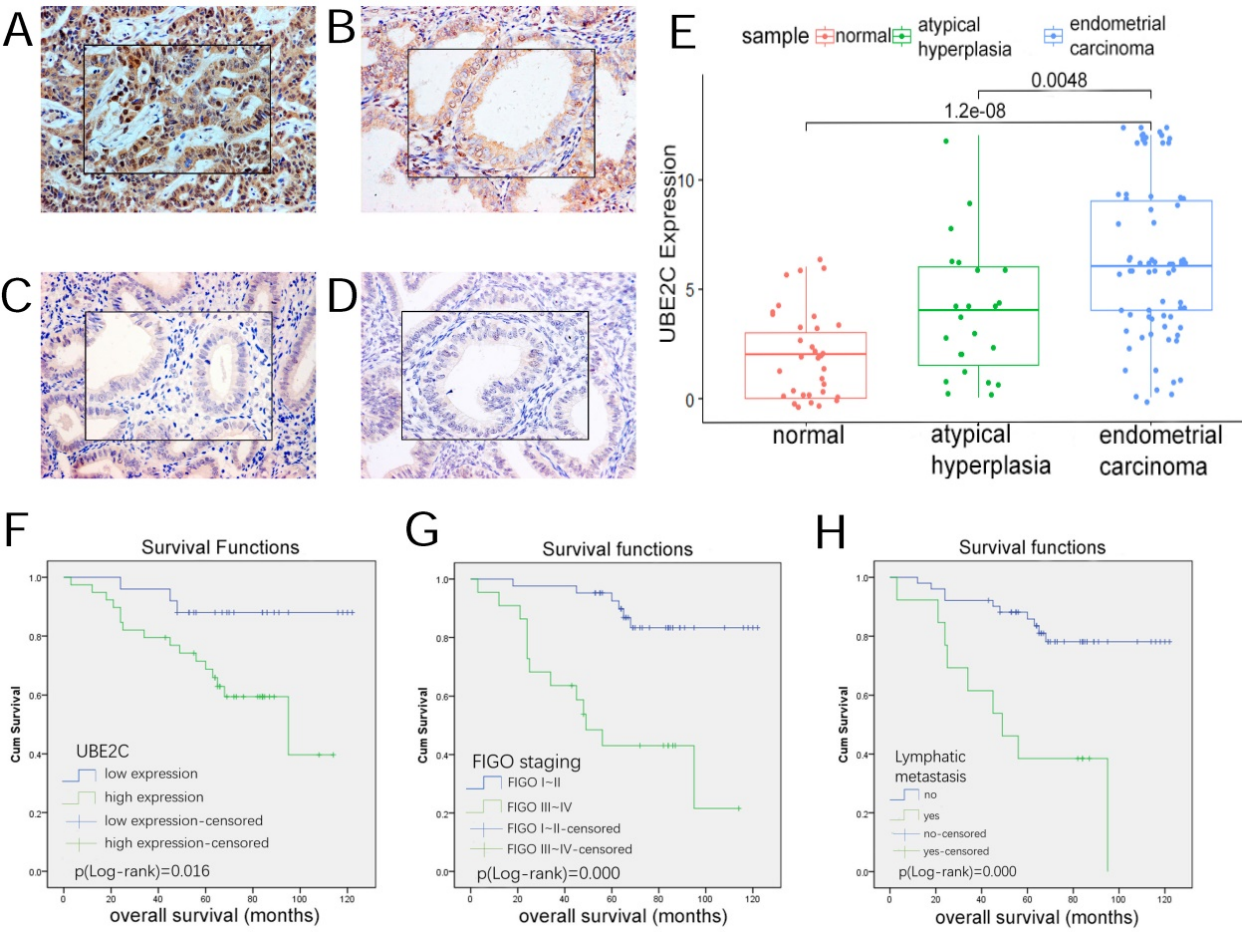
** UBE2C expression in endometrial tissues and its effect on prognosis. (A~D)** Expression of UBE2C in endometrial tissues(SP*200, central*400) A,Endometrial carcinoma B,atypical hyperplasia C,secretory endometrium D,proliferative endometrium. **(E)**boxplot of UBE2C expression in endometrial tissues. **(F~H)** the influence of clinicopathologic parameters on overall survival. F,UBE2C expression. G,FIGO staging. H,lymphatic metastasis.

As Table 7 and Figure 7(A~E) demonstrates, UBE2C expression was detected in each group of our patient samples, with cytoplasm and membrane mainly stained. The positive rate (87.50%) and high expression rate (62.50%) of cancerous tissues were significantly higher than that in endometrial atypical hyperplasia (60.87% and 30.43%) and normal endometrial tissues (35.29% and 11.76% ) (p<0.05).

No significant difference was noted in the expression level between atypical hyperplasia and normal tissues.

#### Correlation of UBE2C expression and clinicopathologic features

In our study, 72 endometrial carcinoma patients were involved. They were divided into two groups, that is, low-UBE2C-expression(-/+) group and high- UBE2C-expression(++/+++) group. As the statistics show in Table 8, the high expression rate in Stage III~IV(88.89%) was significantly higher than that in Stage I~II(53.70%) (p<0.05). Also, the high expression rate of the patients with lymphatic metastasis(92.86%) was significantly higher than that of the patients with no lymphatic metastasis (58.70%) (p<0.05). In terms of histological classification, 8 cases were diagnosed as unknown and no significant correlation was noted in the relationship between the UBE2C expression and histological classification (p>0.05). Although the ≥50%-myometrial-invasion group exhibited a higher expression rate than the <50%-myometrial-invasion group, the difference was not statistically significant (p>0.05).

#### The influence of UBE2C expression on the overall survival

72 endometrial carcinoma patients were followed up for 54-124 months from September 2007 till the end of January 2018, of whom 8 patients were lost follow-up, 9 died of metastasis and recurrence, 10 died of other diseases, and 45 alive. The median survival time was 69 months (3 ~ 122 months).

Kaplan-Meier method and Log-rank test were used to predict the impact of clinicopathologic parameters on the overall survival of the patients. In Figure 7(F~H), low-UBE2C-expression patients had a better prognosis than those with high-UBE2C-expression, which was statistically significant(p<0.05). In addition, the survival time of the patients with advanced FIGO stage III~IV, poor differentiation, lymphatic metastasis, ≥50% myometrial invasion were significantly shorter than those with early FIGO stage I~II, well~moderate differentiation, no lymphatic metastasis and <50% myometrial invasion(p<0.05).

Age and pathological type were not significantly associated with overall survival.

#### Identification of prognostic risk factors

Cox proportional hazard model was built to predict prognostic risk factors. As Table 9 reveals, univariate analysis showed the overall survival was significantly correlated with FIGO staging (p=0.000), UBE2C expression (p=0.006), lymphatic metastasis (p=0.003) and myometrial invasion(p=0.007). Multivariate analysis showed that UBE2C expression (p=0.047), FIGO staging(p=0.000), lymphatic metastasis (p=0.001) and myometrial invasion (p=0.003) were independent risk factors in predicting the prognosis of endometrial carcinoma patients.

### Molecular mechanism of UBE2C

**Figure 8:**
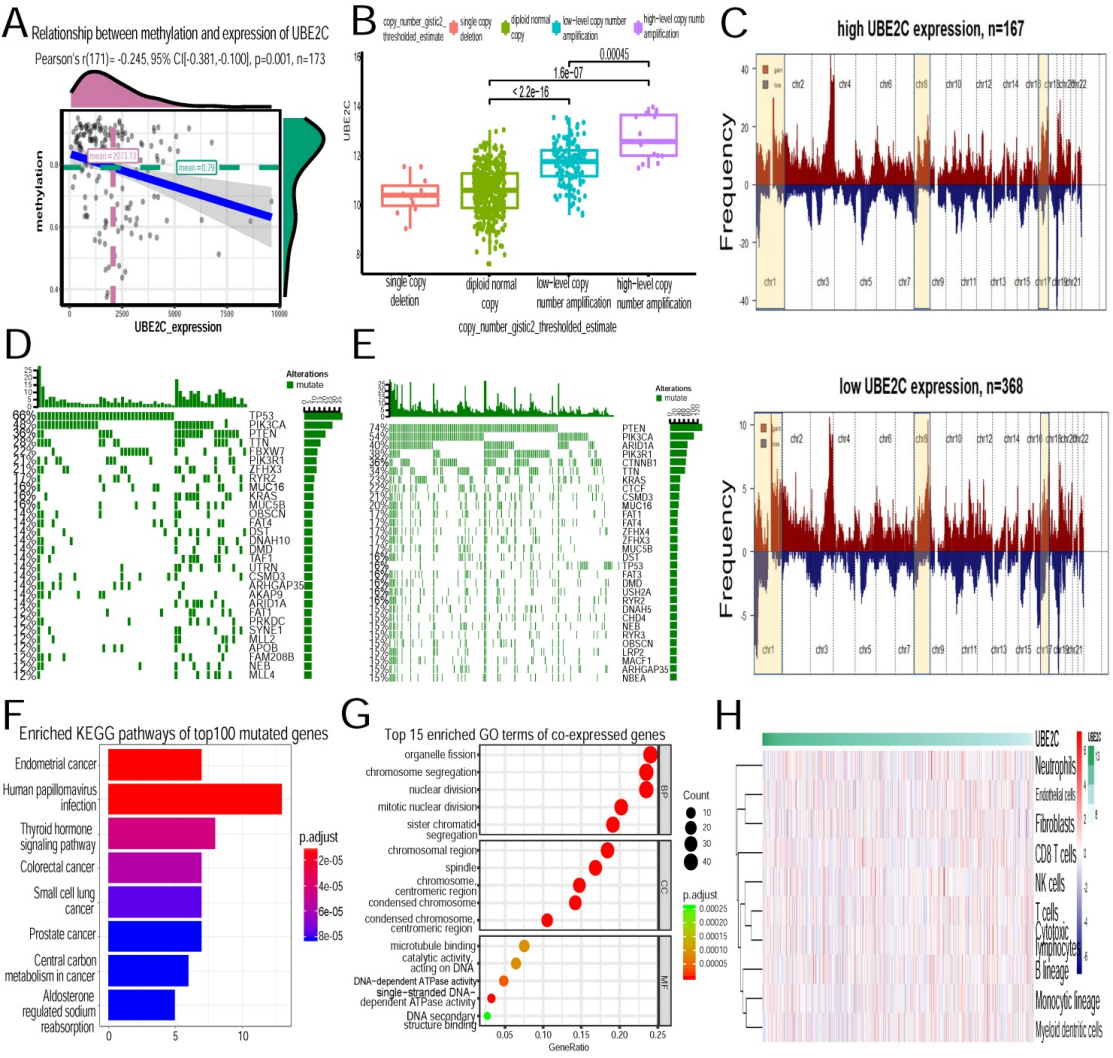
** Molecular mechanisms of high expression of UBE2C. (A)**Relationship between methylation and expression of UBE2C. Methylation of the gene was negatively correlated with its expression(p=0.001). **(B)**Relationship between copy number and expression of UBE2C. Copy number of the gene was positively correlated with its expression(p<0.001).** (C)** Composite copy number profiles for high-UBE2C-expression UCEC and low-UBE2C-expression UCEC with gains in red and losses in blue and yellow highlighting the differences**. (D)**Top 30 somatic mutated genes in high-UBE2C-expression group, green boxes represent mutations in the individual. **(E)** Top 30 somatic mutated genes in low-UBE2C-expression group. **(F)**Enriched KEGG pathways of top 100 mutated genes in high-UBE2C-expression group. **(G)**Top 15 enriched GO terms of co-expressed genes of UBE2C **(H)**Association between UBE2C expression and immune cell populations.

We then dug into the molecular mechanism behind the high expression level of UBE2C in EC patients. As Figure 8A demonstrated, methylation was negatively correlated with UBE2C expression (p=0.001,Pearson's r =-0.245). High level of UBE2C could be partially attributed to hypomethylation. Copy number variation was classified into four levels: single copy deletion, diploid normal copy, low-level copy number amplification and high-level copy number amplification. As copy number increased, the corresponding UBE2C expression elevated(Figure 8B). When we mapped copy number variation onto the entire genome, we found amplification peaks of high-UBE2C-expression group had greater gistic scores and frequency compared to low-UBE2C-expression group(Figure 8C).GISTIC results revealed in high-UBE2C-expression group, amplification peaks appeared in chromosome 1p34.2,1p36.22,1q21.3,1q22,1q32.1,1q42.3,2p23.2,2q13,3p21.1,3p25.1,3q26.2,3q29,4p16.3,6p24.2,6q25.1,7q32.2,8p11.21,8p11.22,8q11.23,8q24.21,10q22.2,11q13.1,11q13.2,12q13.11,12q13.2,16p11.2,17q11.2,17q12,17q21.32,17q25.1,17q25.3,18q11.2,19p13.11,19p13.2,19q12,19q13.2,20q11.21,20q13.12,20q13.33 (residual q value<0.05). While in low-UBE2C-expression group, amplification peaks appeared in 1p34.2, 1p35.2, 1q21.3, 1q22, 2q13, 3p25.1, 3q26.2,3q29,6p24.2,6q25.1,8p11.21,8q11.23,8q21.11,8q24.21,8q24.21,9p24.2,10q22.2,11q13.3,12p12.1,12q13.2,17q11.2,17q12,17q25.1,17q25.3,19p13.12,19p13.2,19p13.2,19q12,20q11.21,20q13.12,20q13.33,22q12.3 (residual q value <0.05). The amplification peaks of the two groups differed in the following loci: 1p36.22, 1q32.1,1q42.3, 2p23.2,3p21.1,4p16.3,7q32.2, 8p11.22, 11q13.1, 11q13.2, 12q13.11, 16p11.2, 17q21.32, 18q11.2, 19p13.11.

In terms of somatic mutations, TP53(66%), PIK3CA(48%), PTEN(36%), TTN(28%), FBXW7(22%), PIK3R1(21%), ZFHX3(21%), RYR2(21%), MUC16 (17%), KRAS(17%) were the top ten genes significantly enriched in the high-expression-UBE2C group. In contrast, PTEN(74%), PIK3CA(54%), ARID1A (40%), PIK3R1(38%), CTNNB1(36%), TTN(34%), KRAS(23%), CTCF(22%), CSMD3(21%), MUC16(20%) were the top ten genes significantly enriched in the low-expression-UBE2C group(Figure 8 D~E). Top 100 mutated genes in high-UBE2C-phenotype were then collected to conduct KEGG pathway analysis. It turned out that they were mainly associated with the activation of the following pathways: endometrial cancer, human papillomavirus infection, thyroid hormone signaling pathway, colorectal cancer and small cell lung cancer (Figure 8F).

GO functional enrichment analysis showed co-expressed genes with UBE2C were mainly involved in organelle fission, chromosome segregation, nuclear division, mitotic nuclear division and sister chromatid segregation (Figure 8G).

Through the Microenvironment Cell Populations-counter method^22^, we evaluated the association between UBE2C and immune cell populations from transcriptomic data. But no strong correlation between UBE2C and T cells, Monocytic lineage and Myeloid dendritic cells was noticed (Figure 8H).

## Discussion

Endometrial carcinoma is the most frequently occurring tumor in female reproductive system. Although postmenopausal bleeding may assist in the detection of EC, a recent systematic review and meta-analysis shows only 9%( 95%CI, 8%-11%) women with postmenopausal bleeding will be diagnosed as EC^4^. According to the SEER (Surveillance, Epidemiology, and End Results) database, the 5-year survival rate of the patients who have distant metastasis falls to 16%^1^. Therefore, it is of great urgency to discover new reliable clinical detection markers for early diagnosis and prognostic prediction of EC.

With the application of high throughout sequencing in clinical studies, large volumes of data are available online. In addition, the exceptional heterogeneity of cancer and individual differences emerge as great obstacles to the diagnosis and precision treatment, which makes it pressing and meaningful to identify new effective biomarkers on a larger scale of data.

It is the first time that our study integrated two endometrial carcinoma GEO datasets, i.e.GSE63678 and GSE17025, and TCGA UCEC mRNA seq data, to screen for the common DEGs. As a result, 344 genes were detected, of which 170 were up-regulated ,174 down-regulated. GO functional enrichment analysis showed the upregulated genes were mainly enriched in mitotic nuclear division (ontology: BP), the spindle (ontology: CC), and microtubule binding (ontology:MF). KEGG pathway analysis demonstrated that the upregulated DEGs were mainly enriched in five pathways, that is, cell cycle(hsa04110), oocyte meiosis(hsa04114), cellular senescence(hsa04218), carbon metabolism (hsa01200), p53 signaling pathway (hsa04115). As is well known, endometrial carcinoma originates from the aberrant growth of the endometrium. The enrichment evidence strongly consolidates the speculation that these DEGs can affect the proliferation and apoptosis of endometrial cancer cells through the aforementioned pathways and thus regulate the onset and progression of EC.

Tumor invasion and metastasis is a complicated pathophysiological process regulated by comple x molecular mechanisms. To elucidate the interaction among DEGs, a PPI network consisting 284 nodes and 3697 edges was constructed. In order to find genes that play significant role in the development of EC, MCODE was used to identify significant module, which was composed of 76 nodes and 2677 edges. Top 20 genes were selected based on degrees in the module to probe into their impacts on the overall survival of EC patients. Consequently, high expression of 15 genes, namely, CCNB2, CDC20, BUB1B, UBE2C, AURKB, FOXM1, NCAPG, RRM2, TPX2, DLGAP5, CDCA8, CDC45, MKI67, BUB1, KIF2C, were associated with poor prognosis (p<0.05).

Given the higher degree and smaller p value (p=0.011), UBE2C was selected for further validation in TCGA cohort and in our patient samples. Of note, our team previously found that UBE2C might be associated with poor prognosis in ovarian cancer patients 23. Therefore, it was speculated this gene might play multiple roles in the tumorigenesis of female reproductive system and its verification could shed some light on the current research.

In TCGA cohort, overexpressed expression of UBE2C in EC was associated with advanced clinical pathologic characteristics (poor differentiation, serous endometrial adenocarcinoma, FIGO stage II~IV, distant metastasis), and poor prognosis. Notably, the expression level of UBE2C in EC tissues was significantly higher than that in non-cancerous tissues. This suggests UBE2C could serve as a potential biomarker for the early detection and diagnosis of EC.

To gain an insight into the UBE2C-involved mechanisms underlying the onset and progression of EC, GSEA was performed. The results showed that high UBE2C expression was associated with the activation of the following pathways: G2M_CHECKPOINT, E2F_TARGETS, MYC_TARGETS_V1, DNA_REPAIR, MYC_TARGETS_V2, SPERMATOGENESIS. The association and the regulatory mechanisms of E2F family of transcription factors with UBE2C, MYC with UBE2C and the roles they play in the carcinogenesis of endometrial carcinoma have not been investigated, which offers new direction for the current research.

UBE2C was first discovered to promote APC (anaphase-promoting complex)-dependent ubiquitination 24. While APC itself could drive the disassembly of checkpoint complexes and the consequent inactivation of the checkpoint, high level of UBE2C observed in tumor cells was likely to promote the process 25-27.

Recently, UBE2C was mainly studied in gastric cancer, non-small cell lung cancer (NSCLC), breast cancer and colorectal cancer. In gastric cancer, Zhang et al.^9^ found that overexpression of UBE2C correlated with advanced clinicopathological parameters and poor prognosis by immunohistochemical staining. Guo et al.^8^ reported deregulation of UBE2C-mediated autophagy repression aggravated NSCLC progression. UBE2C was also highly expressed in breast microcalcification lesions, which is the most common mammographic feature of early breast cancer^28^. Rawat et al.^29^ confirmed inhibition of UBE2C sensitized breast cancer cells to radiation, doxorubicin, tamoxifen and letrozole using colorimetric and clonogenic assays. UBE2C expression was also a predictor of prognosis and sensitivity to the antineoplastic treatment for colorectal cancer patients^30^. However, Kefeli et al.^11^ described a statistically significant difference of UBE2C positivity between the carcinoma group and proliferative endometrium, disordered proliferative endometrium, and nonatypical hyperplasia. But in the malignant group, there was no significant association between UBE2C expression and tumor grade and stage.

Considering prediction of molecular markers from mRNA level was far from perfect 31, we then carried out immunohistochemistry experiment to validate UBE2C expression in our patient samples on protein level. Of note, our samples included atypical hyperplasia tissues, which would make up for the deficiency of GEO and TCGA data. The immunohistochemistry results indicated that the high expression rate in tumor samples was significantly higher than that in atypical hyperplasia and normal tissues(p<0.05). Increased expression of UBE2C was associated with advanced FIGO stage and lymphatic metastasis(both p<0.05). Kaplan-Meier survival curve showed UBE2C overexpression was related with poor prognosis. Cox multivariate analysis demonstrated UBE2C was an independent prognostic risk factor of survival probability. Compared with Kefeli's study, we presumed the inconsistence of the two studies lied in the quantity of the samples. In Kefeli's study, only 32 EC tissues(stage I 25 cases, stage II 3 cases, stage III 4cases, no stage IV) were involved. It might account for the difference between the two findings. Although our study involved 72 EC patients, the quantity was still limited and a study on larger scale of samples is also required for validation.

Then we tried to dig up the molecular mechanisms behind the high expression of UBE2C. TCGA cohort was divided into two groups according to the UBE2C expression, and somatic mutations and copy number variation were investigated separately. In high-UBE2C-expression phenotype, TP53, TTN, FBXW7, ZFHX3, RYR2 were significantly enriched. PIK3CA (48% vs 54%),PTEN(36% vs 74%), PIK3R1 (21% vs 38%), MUC16 (17% vs 20%), KRAS (17% vs 23%) were differentially enriched in high- and low- UBE2C-expression groups. As TP53 was the top one mutated gene in high-UBE2C-expression(66% vs 16% in low-UBE2C-expression), it might play contributive role in the high level of UBE2C.KEGG pathway analysis of top 100 mutated genes in high-UBE2C- phenotype revealed they were mainly significantly enriched in endometrial cancer, human papillomavirus infection, thyroid hormone signaling pathway, colorectal cancer and small cell lung cancer. Based on the results, we suspect those mutated genes take effect in the overexpression of UBE2C and finally promote the onset and progression of EC. In addition to this, the relationship between copy number variation and UBE2C expression was then analyzed. It turned out that as the copy number amplification increased, the corresponding expression of UBE2C significantly increased too. To get more understanding of the impact of high level of UBE2C on the entire genome, copy number segments of TCGA after removing germline cnv were analyzed by GISTIC2 software. There were 43 focal amplification peaks in high- UBE2C-phenotype, in contrast to 32 peaks in low-UBE2C-phenotype. The two groups had the following distinct sites: 1p36.22, 1q32.1,1q42.3, 2p23.2, 3p21.1, 4p16.3, 7q32.2, 8p11.22, 11q13.1, 11q13.2, 12q13.11, 16p11.2, 17q21.32, 18q11.2, 19p13.11. Notably, high-UBE2C-phenotype group had greater gistic scores and higher frequency of almost every amplification peaks, and this may provide new prospective into the progression of endometrial cancer.

DNA methylation, as epigenetic modification mechanism, regulates gene expression by acting with transcription factors or linking to chromosomal instability. To explore more about the mechanisms of high level of UBE2C, our study found DNA hypomethylation of UBE2C correlated negatively with its expression. Kamalakaran et al.^32^ discovered UBE2C exhibited expression-methylation correlation and had prognostic values in breast cancer. Based on what we have discussed before, we assume TP53 mutation, elevated copy number, hypomethylation all contribute to the overexpressed UBE2C and UBE2C hypomethylation and its elevated mRNA expression are indicators of poor prognosis in EC patients.

To clarify the biological role of UBE2C in EC, top 200 co-expressed genes of UBE2C downloaded from cBioportal were analyzed by GO functional enrichment analysis. Biological process indicated that organelle fission, chromosome segregation, nuclear division, mitotic nuclear division and sister chromatid segregation were significantly enriched. The molecular function of UBE2C was mainly enriched in microtubule binding, catalytic activity and DNA-dependent ATPase activity. Consequently, we infer UBE2C and its relevant genes may act on DNA duplication and then affect the cell cycle, proliferation and apoptosis of endometrial carcinoma cells. Till now, no research has focused on the specific role of UBE2C in the progression of EC, and this will point new direction for the current studies of EC.

In summary, our study combined two GEO datasets and TCGA UCEC data to screen for key genes involved in the progression of EC, one of which was UBE2C. Survival probability and the relationship of its expression and clinicopathologic parameters were analyzed in TCGA cohort on mRNA level and in our patient samples on protein level by immunohistochemistry. Genomic alterations and biological role of UBE2C were investigated to explore the potential mechanisms underlying its overexpression in oncogenesis of endometrial carcinoma. Our future study will address the biological behavior and molecular mechanism of UBE2C in EC by cytologic experiments, which will greatly advance our understanding and provide better implications for treating patients with efficacy.

## Supplementary Material

Table S1: DEGs of GSE63678.

Table S2: DEGs of GSE17025.

Table S3: DEGs of TCGA-UCEC.

## Figures and Tables

**Table 1:** Details of the datasets

Dataset		sample	normal	tumor	platform	reference
GEO	GSE63678	endometrium	5	7	GPL571	Pappa et al.(2015)
	GSE17025	endometrium	12	91	GPL570	Day et al.(2009)
TCGA-UCEC		endometrium	23	543	-	-

**Table 2:** Our endometrial carcinoma patient characteristics

Clinical characteristics		total	%
**Age at diagnosis**		58(36-79)	
**histological classification**	well	16	22.2%
	moderate	24	33.3%
	poor	24	33.3%
	unknown	8	11.1%
**histopathology**	endometroid adenocarcinoma	37	51.4%
	serous carcinoma	22	30.6%
	clear cell carcinoma	8	11.1%
	mucinous carcinoma	5	6.94%
**FIGO cancer staging system**	Stage I	48	66.7%
	Stage II	6	8.33%
	Stage III	15	20.8%
	Stage IV	3	4.17%
**Lymphatic metastasis**	yes	14	19.44%
	no	46	63.89%
	unknown	12	16.67%
**Myometrial invasion**	<50%	47	65.3%
	≥50%	25	34.7%

†FIGO: International Federation of Gynecology & Obstetrics

**Table 3:** Top 15 GO enrichment terms of the upregulated and downregulated genes.

Category	ID	Description	Count	P value
**A.** Top 15 enriched GO terms of upregulated genes				
BP	GO:0140014	mitotic nuclear division	38	5.13E-34
BP	GO:0007059	chromosome segregation	41	5.83E-33
BP	GO:0000280	nuclear division	43	6.29E-33
BP	GO:0000819	sister chromatid segregation	35	7.06E-32
BP	GO:0000070	mitotic sister chromatid segregation	30	1.99E-31
CC	GO:0005819	spindle	34	1.54E-26
CC	GO:0000793	condensed chromosome	24	1.94E-19
CC	GO:0000775	chromosome, centromeric region	23	2.35E-19
CC	GO:0000779	condensed chromosome, centromeric region	19	8.44E-19
CC	GO:0072686	mitotic spindle	17	7.54E-18
MF	GO:0008017	microtubule binding	15	3.09E-08
MF	GO:0003777	microtubule motor activity	10	1.62E-07
MF	GO:0015631	tubulin binding	16	1.80E-07
MF	GO:0035173	histone kinase activity	5	5.05E-07
MF	GO:0003774	motor activity	10	6.04E-07
		
**B.** Top 15 enriched GO terms of downregulated genes				
BP	GO:0048639	positive regulation of developmental growth	11	8.69E-07
BP	GO:0070838	divalent metal ion transport	17	1.31E-06
BP	GO:0072511	divalent inorganic cation transport	17	1.48E-06
BP	GO:0006816	calcium ion transport	16	1.57E-06
BP	GO:0090287	regulation of cellular response to growth factor stimulus	13	1.60E-06
CC	GO:0031012	extracellular matrix	16	9.36E-06
CC	GO:0005578	proteinaceous extracellular matrix	13	4.54E-05
CC	GO:0005901	caveola	6	8.25E-05
CC	GO:0044853	plasma membrane raft	6	0.00029
CC	GO:0014704	intercalated disc	4	0.001145
MF	GO:0005262	calcium channel activity	8	1.42E-05
MF	GO:0005518	collagen binding	6	2.31E-05
MF	GO:0005539	glycosaminoglycan binding	10	2.85E-05
MF	GO:0015085	calcium ion transmembrane transporter activity	8	4.55E-05
MF	GO:0070679	inositol 1,4,5 trisphosphate binding	3	0.000199

†**Note**: BP: biological process. CC: cellular component. MF: molecular function

**Table 4:** Top 10 KEGG pathways of the upregulated and downregulated genes.

ID	Description	GeneRatio	P value	Count
**A.** Top 10 KEGG pathways of upregulated genes				
hsa04110	Cell cycle	16/95	2.24E-12	16
hsa04114	Oocyte meiosis	11/95	4.34E-07	11
hsa04218	Cellular senescence	11/95	5.04E-06	11
hsa01200	Carbon metabolism	9/95	1.46E-05	9
hsa04115	p53 signaling pathway	7/95	3.20E-05	7
hsa04914	Progesterone-mediated oocyte maturation	8/95	3.33E-05	8
hsa00240	Pyrimidine metabolism	5/95	0.000738334	5
hsa05170	Human immunodeficiency virus 1 infection	9/95	0.001414968	9
hsa05164	Influenza A	8/95	0.00141817	8
hsa00010	Glycolysis / Gluconeogenesis	5/95	0.001647227	5
				
**B.** Top 10 KEGG pathways of downregulated genes				
hsa01521	EGFR tyrosine kinase inhibitor resistance	6/81	0.000198	6
hsa04630	JAK-STAT signaling pathway	8/81	0.000342	8
hsa04020	Calcium signaling pathway	8/81	0.000857	8
hsa05218	Melanoma	5/81	0.001042	5
hsa05206	MicroRNAs in cancer	10/81	0.001357	10
hsa04726	Serotonergic synapse	6/81	0.001472	6
hsa04022	cGMP-PKG signaling pathway	7/81	0.001843	7
hsa04923	Regulation of lipolysis in adipocytes	4/81	0.002675	4
hsa05215	Prostate cancer	5/81	0.003892	5
hsa04151	PI3K-Akt signaling pathway	10/81	0.004645	10

**Table 5:** UBE2C expression^a^ associated with clinicopathological characteristics (binary logistic regression)

Clinical characteristics		Total	Odds ratio in UBE2C expression	p-value
**Stage**				
	II vs I	390	1.898(1.028-3.504)	0.041*
	III vs I	463	1.735(1.119-2.691)	0.014*
	IV vs I	368	3.152(1.462-6.796)	0.003*
**Histologic grade**				
	Moderate vs well	218	1.632(0.693-3.843)	0.262
	Poor vs well	423	7.578(3.689-15.565)	0.000*
	Poor vs moderate	445	4.643(2.657-8.112)	0.000*
**Histology**				
	SEA vs EEA	521	4.813(3.103-7.465)	0.000*
	SEA vs MSE	136	3.055(1.156-8.071)	0.024*
**Cancer status**				
	With tumor vs tumor free	523	2.548(1.623-3.998)	0.000*
**New neoplasm event**				
	Distant metastasis vs no	475	4.043(1.299-12.584)	0.016*
	Locoregional recurrence vs no	485	3.285(1.406-7.676)	0.006*
**Myometrial invasion**				
	≥50 vs <50	470	1.392(0.933-2.078)	0.105
**Peritoneal wash**				
	Positive vs negative	407	1.981(1.113-3.525)	0.020*

**Table 6:** Gene sets enriched in phenotype High

MSigDB collection	Gene set name	NES	NOM p-value	FDR q-value
h.all.v6.2.symbols. gmt[Hallmarks]	HALLMARK_G2M_CHECKPOINT	1.95	0.000	0.013
	HALLMARK_E2F_TARGETS	1.91	0.000	0.010
	HALLMARK_MYC_TARGETS_V1	1.90	0.014	0.008
	HALLMARK_DNA_REPAIR	1.88	0.002	0.007
	HALLMARK_MYC_TARGETS_V2	1.83	0.008	0.010
	HALLMARK_SPERMATOGENESIS	1.57	0.004	0.090

† NES: normalized enrichment score; NOM: nominal; FDR: false discovery rate. Gene sets with NOM p-value <0.05 and FDR q-value <0.25 were considered as significant.

**Table 7:** Expression of UBE2C in endometrial tissues

group	cases	UBE2C staining					
		low(-/+)		high(++/+++)			
		-	+	++	+++	positive rate	high expression rate
normal	34	22	8	4	0	35.29%	11.76%
atypical hyperplasia	23	9	7	5	2	60.87%	30.43%
cancerous tissues	72	9	18	21	24	87.50%*	62.50%*

Note: * means the UBE2C expression was significantly higher than that in atypical hyperplasia and in normal tissues(p<0.05).

**Table 8:** UBE2C expression associated with clinicopathological features

parameter	cases	UBE2C staining					
		low(-/+)		high(++/+++)			
		-	+	++	+++	high expression rate	p value
**histological classification**							
well-moderate	40	4	11	12	13	62.50%	p>0.05
poor	24	3	6	6	9	62.50%	
unknown	8	2	1	3	2	62.50%	
**FIGO staging**							
Stage I,II	54	7	18	13	16	53.70%	p<0.05*
Stage III,IV	18	2	0	8	8	88.89%	
**histopathology**							
endometroid adenocarcinoma	37	2	9	14	12	70.27%	p>0.05
serous carcinoma	22	6	3	5	8	59.09%	
clear cell carcinoma	8	1	3	0	4	50%	
mucinous carcinoma	5	0	3	2	0	40%	
**myometrial invasion**							
<50%	47	7	13	12	15	57.45%	p>0.05
≥50%	25	2	5	9	9	72.00%	
**lymphatic metastasis**							
no	46	5	14	12	15	58.70%	p<0.05*
yes	14	1	0	7	6	92.86%	
unknown	12	3	2	4	3	58.33%	

†FIGO: International Federation of Gynecology & Obstetrics

**Table 9:** Cox regression analysis of overall survival in our patient samples

parameter	univariate analysis			multivariate analysis		
	HR	95% CI of HR	p-value	HR	95%CI of HR	p-value
age (≥60vs<60)	2.496	0.999-6.235	0.050	0.845	0.301-2.376	0.750
histological classification (poor vs well-moderate)	2.516	0.990-6.394	0.053	1.392	0.467-4.151	0.553
FIGO staging (III~IV vs I~II)	8.288	3.231-21.258	0.000*	7.793	1.917-31.670	0.000*
UBE2C expression (high vs low)	7.913	1.823-34.355	0.006*	5.034	1.020-24.836	0.047*
lymphatic metastasis (yes vs no)	4.090	1.640-10.198	0.003*	0.067	0.014-0.327	0.001*
myometrial invasion (≥50% vs <50%)	3.486	1.399-8.681	0.007*	5.248	1.739-15.834	0.003*

† CI, confidence interval; FIGO, International Federation of Gynecology and Obstetrics; HR, hazard ratio

## Abbreviations

EC: Endometrial carcinoma
GEO: Gene Expression Omnibus
TCGA: The Cancer Genome Atlas
DEGs: differentially expressed genes
GO: gene ontology
KEGG: Kyoto Encyclopedia of Gene and Genome
PPI: protein-protein interaction
UBE2C: Ubiquitin Conjugating Enzyme E2 C
FIGO: International Federation of Gynecology and Obstetrics
UCEC: uterine corpus endometrial carcinoma
STRING: Search Tool for the Retrieval of Interacting Genes
CNV: copy number variation
GISTIC: Genomic Identification of Significant Targets in Cancer
DAVID: The Database for Annotation, Visualization and Integrated Discovery
MCODE: Molecular Complex Detection
GSEA: Gene set enrichment analysis
BP: biological process
MF: molecular function
CC: cellular component
OR: odds ratio
HR: hazard ratio
